# Translation and Validation of the Bulgarian Version of the Boston Carpal Tunnel Questionnaire

**DOI:** 10.7759/cureus.10901

**Published:** 2020-10-11

**Authors:** Vesselin Karabinov, Svetoslav A Slavchev, Georgi P Georgiev

**Affiliations:** 1 Neurology, National Cardiology Hospital, Sofia, BGR; 2 Orthopaedics and Traumatology, Medical University of Sofia, Sofia, BGR

**Keywords:** boston carpal tunnel questionnaire, bctq, translation, validation, bulgarian

## Abstract

Background and Objective: Carpal tunnel syndrome (CTS) is the most common mononeuropathy in humans. Despite the continuous improvement of diagnostic and treatment methods, difficulties remain in the evaluation and quantification of such symptoms as pain, paresthesias, hypesthesia, and hyperesthesia. Numerous tests and questionnaires have been developed for patients with upper limb disease, but the most specific for CTS and the most commonly used is the Boston Carpal Tunnel Questionnaire (BCTQ). BCTQ has been translated and validated for use in many languages, but there is no valid version in Bulgarian yet. The purpose of this work is to create and validate a Bulgarian version of BCTQ, with a recommendation for its use in Bulgarian patients.

Methods: The process was divided into two parts. The first part included a translation and a cultural-linguistic adaptation of the Bulgarian version of BCTQ. In the second part, verification of the psychometric properties of the Bulgarian BCTQ, we investigated the reliability, validity and responsiveness of the Bulgarian version of BCTQ. We evaluated BCTQ's construct validity by comparing its results with the score of the Disabilities of the Arm, Shoulder and Hand (DASH) questionnaire. The study was performed on a group of 64 patients with a confirmed diagnosis of CTS. All patients were evaluated using the BCTQ and DASH questionnaires. A subgroup of 26 patients underwent open surgical decompression of the carpal canal using a mini-incision technique. The subgroup was evaluated through BCTQ and DASH questionnaires preoperatively and through BCTQ postoperatively at the third month after the intervention.

Results: In the first part of the study, the final version of the questionnaire was presented. Cronbach’s alpha coefficient was 0.88 for the Symptom Severity Scale (SSS) and 0.87 for the Functional Status Scale (FSS). The reproducibility of scores showed an extremely high degree of correlation between the two consecutive BCTQ scores at one-week interval (SSS r=0.99, p<0.0001; FSS r=1, p<0.0001). The criterion validity of the Bulgarian version of BCTQ revealed a strong correlation between the results of the BCTQ and the DASH questionnaires. (SSS r(62)=0.569, p<0.00001; FSS r(62)=0.605, p<0.00001). There was a statistically significant decrease in BCTQ results after surgery for both the SSS (t=-9.43, p<0.00001) and the FSS (t=-9.82, p<0.00001).

Conclusion: Our study created a translated and culturally adapted version of BCTQ. The Bulgarian version of BCTQ is reliable, valid, and responsive for measuring symptoms and functional deficits in patients with CTS.

## Introduction

The carpal tunnel syndrome (CTS) is the most common musculoskeletal disease in the European countries, the United States, and Canada and in recent years the share of CTS in the structure of musculoskeletal disorders associated with the workplace has increased [1]. Its socio-economic importance is high as it affects people of working age and leads to severe impairment of hand function and quality of life. There are still controversies in the qualitative and quantitative evaluation of subjective sensory symptoms, e.g. pain and paresthesia. Sensory symptoms are at the base of the diagnosis and follow-up of the treatment effect in CTS [2]. Numerous tests and questionnaires have been developed for patients with upper limb disease but the most frequently used disease-specific instrument for CTS is the Boston Carpal Tunnel Questionnaire (BCTQ). The questionnaire was developed by Levine et al. in 1993 [3]. Its creation was dictated by the fact that instrumental methods were predominantly used in the postoperative follow-up of CTS patients while patients themselves perceived greater importance in the changes in sensory symptoms and hand function.

The BCTQ questionnaire consists of two parts - a Symptom Severity Scale (SSS) and a Functional Status Scale (FSS). The Symptom Severity Scale comprises 11 questions and the FSS comprises eight questions, each question scoring from one (no symptoms) to five (very severe symptoms) on a Likert scale. The total score of each scale is obtained by calculating the arithmetic mean of the answers. For both scales, the higher score corresponds to more severe symptoms. The questionnaire is completed by the patient, does not take more than 10 minutes, and does not burden the doctor and the patient.

BCTQ has been translated and validated for use in many languages [4-9], but there is no valid version in Bulgarian yet. The purpose of this work is to create and validate a Bulgarian version of BCTQ, with a recommendation for its use in Bulgarian patients.

## Materials and methods

**Figure 1:**
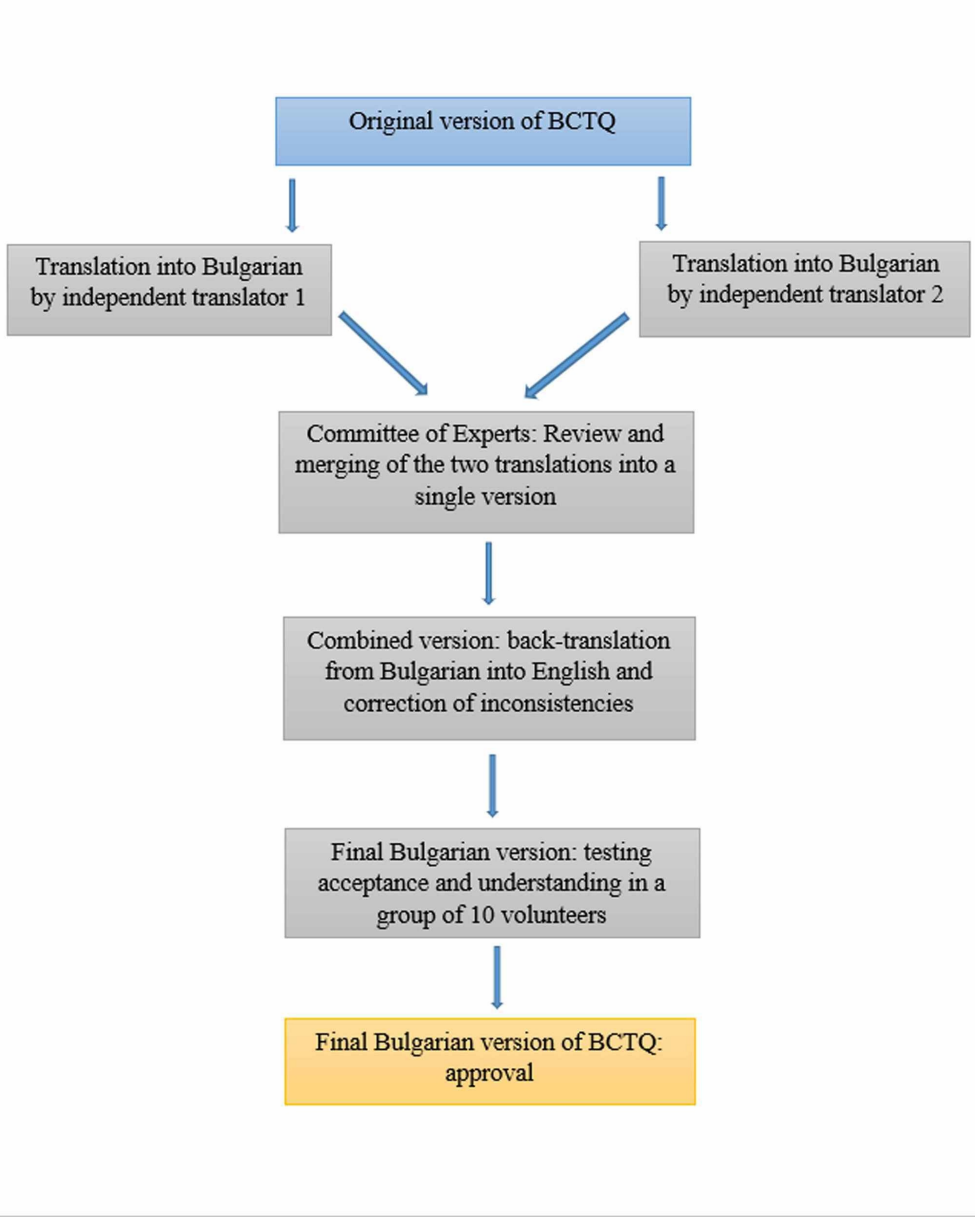
Schematic workflow of the translation of the Bulgarian version of the Boston Carpal Tunnel Questionnaire.

The study was divided into two parts. The first part included a translation from the original English version into Bulgarian and cultural and linguistic adaptation of the Bulgarian version. For this purpose, the original text was translated from English to Bulgarian by two independent translators. The two versions were reviewed and merged into a single version by a committee of experts, including a neurologist, a neuropsychologist, an orthopedist, and a linguist. The resulting questionnaire was back-translated from Bulgarian into English to elucidate any inconsistencies. The final Bulgarian version was checked for acceptance and understanding by testing 10 volunteers (Figure 1).

After the approval of the final version of the questionnaire in Bulgarian, the second part was performed - verification of the psychometric properties of the Bulgarian BCTQ. In this study, we investigated the validity, reliability, and responsiveness of the Bulgarian BCTQ. The study was performed on a group of 64 patients with a confirmed diagnosis of CTS. The criteria for diagnosis and inclusion in the group were history data (burning and predominantly night pain associated with tingling and numbness affecting the thumb, index and middle fingers, and the radial aspect of the ring finger), positive clinical tests (hyperesthesia and two-point discrimination test) and a positive electrodiagnostic study, ability to read and understand the questionnaire, and signed informed consent. The exclusion criteria were: non-native speakers of Bulgarian, illiterate, and mentally disabled patients. All patients were evaluated using the BCTQ and DASH questionnaires. A subgroup of 26 patients underwent open surgical decompression of the carpal canal using a mini-incision technique. The mini-incision subgroup was evaluated through BCTQ and DASH preoperatively and BCTQ postoperatively at the third month after the intervention.

The reliability of the questionnaire was evaluated through its internal consistency by measuring Cronbach's alpha coefficient, with an acceptable value of 0.8, and its reproducibility was evaluated by retesting all patients twice with an interval of one week.

We evaluated BCTQ's construct validity by comparing its results with the score of the Disabilities of the Arm, Shoulder and Hand (DASH) questionnaire. We chose it as an external criterion because of its well-established psychometric properties [10]. This is a patient-reported questionnaire that consists of 30 main items and eight optional items. The DASH can be used to evaluate the function of the upper limb and to track its changes over time [11]. A Bulgarian version of the questionnaire is provided by The Institute for Work & Health, Toronto, Canada.

BCTQ responsiveness was evaluated in a subset of 26 patients by comparing the results of the preoperative administration of the questionnaire and the second administration three months after decompression of the median nerve. To do this, we used statistical hypothesis testing using t-test.

Statistical significance was accepted for p<0.01. The obtained data were summarized and analyzed through Microsoft Excel 2010. Statistica software (Dell Software Inc., Round Rock, TX, USA) was used for the statistical analysis.

## Results

**Figure 2:**
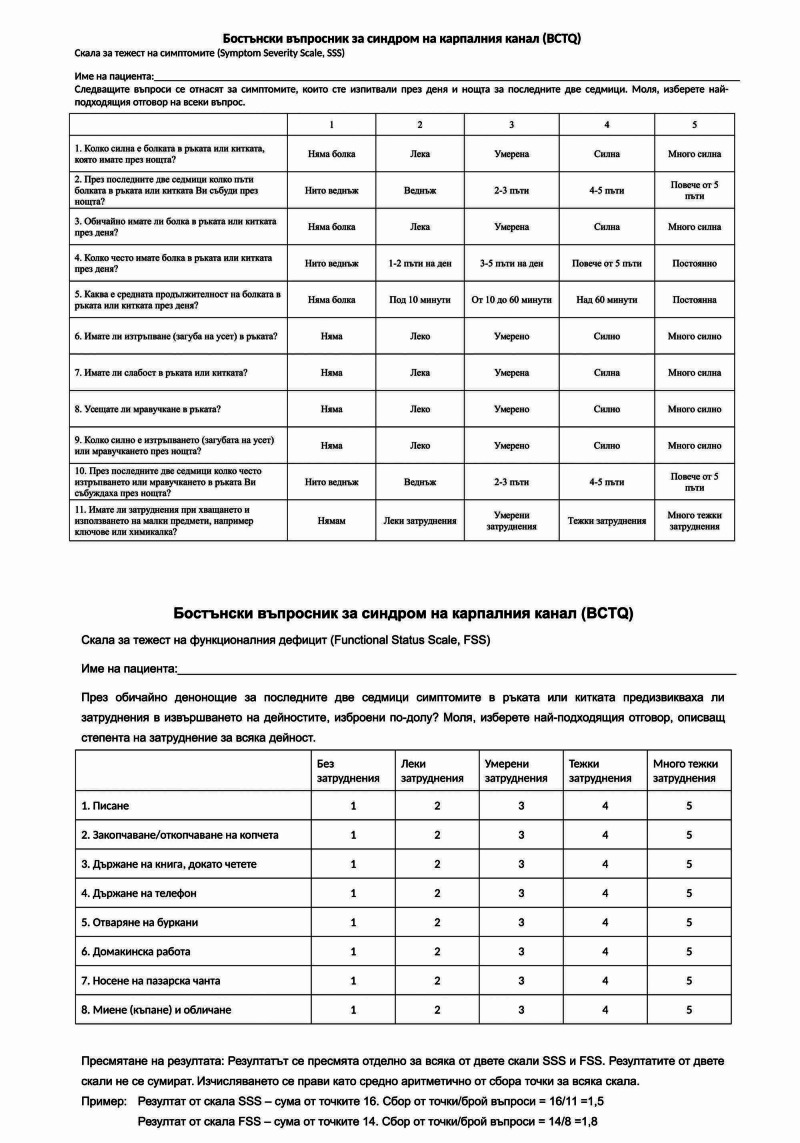
The Bulgarian version of Boston Carpal Tunnel Questionnaire.

After carrying out the translation, cultural and linguistic adaptation, volunteer testing and reflecting comments, the final version of the questionnaire was presented (Figure 2).

The Bulgarian version of the BCTQ scale was self-administered by a group of 64 patients. In our study, women were 76%, respectively 24% of the participants were men, the average age of the participants was 56.7 years (47-66 years). Normal distributions were found for all scores. The data obtained from the BCTQ and DASH questionnaires are presented in Table 1.

**Table 1:** Mean values, standard deviation, and upper and lower limits of the results of the two questionnaires. BCTQ: Boston Carpal Tunnel Questionnaire, DASH: Disabilities of the Arm, Shoulder and Hand, SSS: Symptom Severity Scale, FSS: Functional Status Scale

n=64	Mean	SD	Min	Max
BCTQ SSS	3.14	0.77	1.91	4.64
BCTQ FSS	2.96	0.75	1.38	4.38
DASH	52.73	21.9	13.39	88.33

Cronbach’s alpha coefficient was 0.88 for the Symptom Severity Scale and 0.87 for the FSS, demonstrating a sufficiently high degree of internal consistency for BCTQ. The procedure for sequential exclusion of each element from the scale shows how Cronbach’s alpha would change if each element was excluded from the overall analysis. This procedure also showed a high uniformity of the scale elements since the total value of Cronbach’s alpha changed slightly (Table 2).

**Table 2:** Cronbach's alpha values for the Symptom Severity Scale and the Functional Status Scale with each of the elements excluded. BCTQ: Boston Carpal Tunnel Questionnaire, SSS: Symptom Severity Scale, FSS: Functional Status Scale

BCTQ SSS		BCTQ FSS	
Items	Cronbach’s alpha	Items	Cronbach’s alpha
All itеms	0.88	All itеms	0.87
Q1 excluded	0.87	Q1 excluded	0.89
Q2 excluded	0.88	Q2 excluded	0.84
Q3 excluded	0.87	Q3 excluded	0.86
Q4 excluded	0.86	Q4 excluded	0.85
Q5 excluded	0.86	Q5 excluded	0.84
Q6 excluded	0.87	Q6 excluded	0.85
Q7 excluded	0.88	Q7 excluded	0.85
Q8 excluded	0.87	Q8 excluded	0.85
Q9 excluded	0.87		
Q10 excluded	0.86		
Q11 excluded	0.89		

The reproducibility of scores, as determined by Pearson's correlation coefficient, showed an extremely high degree of correlation between the two consecutive BCTQ scores at a one-week interval (SSS r=0.99, p<0.0001; FSS r=1, p<0.0001). This means that after the control interval, the respondents' estimates have not changed, i.e. the Bulgarian version of BCTQ is resistant to time factor error.

The criterion validity of the Bulgarian version of BCTQ assessed through Pearson’s correlation coefficient, revealed a strong correlation between the results of the BCTQ and the DASH questionnaires. This applies both to the SSS (r(62)=0.569, p<0.00001) and to the FSS (r(62)=0.605, p<0.00001). The results confirm the high level of criterion validity of the Bulgarian version of BCTQ.

To determine the responsiveness of the BCTQ questionnaire, we used t-test, testing the null-hypothesis that there was no significant decrease in BCTQ results after surgery. The results showed a statistically significant difference for both the SSS (t=-9.43, p<0.00001) and the FSS (t=-9.82, p<0.00001). This confirms the ability of the questionnaire to detect objective changes in the patients' condition.

## Discussion

CTS is the most reported nerve compression syndrome, with an incidence of 125-515/100,000, that affects between 2% and 5% of the population. Commonly, CTS is idiopathic but it could also be provoked by tumors, vascular abnormalities, abnormal ligamentous attachments, and different anomalous muscles [12,13]. It is more common in women and mainly affects the dominant hand. In the US, it affects one in 20 people from 40 to 65 years of age [1]. CTS accounted for 0.2% of all hospital visits in the United States in 2006 [14]. There is still no reliable data for Bulgaria. Using a CTS-specific tool with good psychometric properties could accurately assess the patient's condition and measure the effect of treatment. This study examines the psychometric properties of the Bulgarian version of BCTQ. The results show that the Bulgarian version of BCTQ has good validity, reliability, and responsiveness in patients with CTS.

The Bulgarian version of BCTQ showed a high reliability index, expressed by Cronbach's alpha coefficient and reproducibility of results. Cronbach’s alpha values reported by other authors differ, but as in our study, they are above 0.8 for both scales [4-9,15]. The degree of reproducibility of the results in our study exceeds that of many other studies; the Russian version has a coefficient of 1 [15].

The study of criterion validity using the translated and validated DASH questionnaire is widely used in the validation of BCTQ [7,15,16]. In all of them, as in our study, a strong or very strong correlation has been found between the scores obtained from the two questionnaires. In the validation process, BCTQ testing before and after surgery was used by some authors [5,7,15] They reported a decrease in SSS and FSS results from the postoperative study. Lue et al. [7] and Yusupova et al. [15] reported a statistically significant decrease in postoperative results of both scales, demonstrating the responsiveness of BCTQ and its ability to account for changes in patients' clinical status. Our subgroup of operated patients had a statistically significant decrease in the results on both scales, which proves the responsiveness of the Bulgarian version of BCTQ.

## Conclusions

Our study created a translated and culturally adapted version of BCTQ. The Bulgarian version of BCTQ is reliable, valid, and responsive for measuring symptoms and functional deficits in patients with CTS.

The implementation of this validated version will assist healthcare professionals in both research and daily work with CTS patients. Finally, the Bulgarian version of the questionnaire provides a reliable tool for monitoring the status of Bulgarian patients and for evaluating therapeutic strategies and their outcomes.
